# Evaluation of Low-Toxic Hybrid Sol-Gel Coatings with Organic pH-Sensitive Inhibitors for Corrosion Protection of AA2024 Aluminium Alloy

**DOI:** 10.3390/gels9040294

**Published:** 2023-04-02

**Authors:** Eva Jaldo Serrano, Jesús López-Sánchez, Federico García-Galván, Aida Serrano, Óscar Rodríguez de la Fuente, Violeta Barranco, Juan Carlos Galván, Noemí Carmona

**Affiliations:** 1 Departamento de Física de Materiales, Facultad de Ciencias Físicas, Universidad Complutense de Madrid, Plaza Ciencias SN, 28040 Madrid, Spain; 2 Instituto de Cerámica y Vidrio (ICV-CSIC), C/Kelsen 5, Campus de Cantoblanco, 28049 Madrid, Spain; 3 Department of Engineering, School of Architecture, Engineering and Design, Universidad Europea de Madrid, 28670 Villaviciosa de Odón, Spain; 4 Instituto de Magnetismo Aplicado (IMA) “Salvador Velayos”, A6 Km 22,5, 28230 Las Rozas Madrid, Spain; 5 Centro Nacional de Investigaciones Metalúrgicas (CENIM-CSIC), Avda. Gregorio del Amo 8, 28040 Madrid, Spain

**Keywords:** sol-gel, corrosion inhibitors, protective coating, sensor, chlorophenol red, AA2024 aluminium alloy

## Abstract

Today’s environmental needs require the reduction of the weight of vehicles, thus reducing fuel consumption and associated emissions. For this reason, the use of light alloys is being studied, which, due to their reactivity, must be protected before use. In this work, the effectiveness of a hybrid sol-gel coating doped with various organic environmentally friendly corrosion inhibitors applied to an AA2024 lightweight aluminium alloy is evaluated. Some of the inhibitors tested are pH indicators, acting as both corrosion inhibitors and optical sensors for the surface of the alloy. Samples are subjected to a corrosion test in a simulated saline environment and characterised before and after the test. The experimental results regarding their best inhibitor performance for their potential application in the transport industry are evaluated.

## 1. Introduction

The aerospace, automotive, and marine industries have probably made the greatest anti-corrosion efforts in the last two decades, producing advances that have focused on the substitution of various materials mainly for lightweighting, resulting in fuel and CO_2_ emission reductions. In addition, an effort is being made to use more corrosion-resistant materials, better protective finishes and improvements in protective coatings [1].

Light alloys based on aluminium and magnesium are strong candidates for reducing the density of materials used in the transport industry. Their advantages include versatility in production and forming [2,3,4,5,6], a good mechanical strength-to-weight ratio [7], good formability, high fatigue strength [8], and a wide range of possible applications [9]. However, these alloys are very prone to corrosion phenomena, which occur naturally by reacting with oxygen and other gases or ions present in the environment to achieve a state of thermodynamic equilibrium of the material that reduces the internal energetics of the system. In the case of aluminium alloys, the corrosion process is frequent due to the fact that it is a highly reactive element and its alloys contain intermetallic compounds, which can aggravate corrosion phenomena, as they have a different electrochemical potential than the metallic substrate [10,11].

The most common solution used in the past for the protection of metal surfaces was the application of chromium plating treatments, which achieve a good surface finish and improve corrosion behaviour. However, due to the toxicity of hexavalent chromium, international regulations have prohibited its use for some years [12]. Recently, various methods such as painting, lacquering, galvanising and anodising are being developed and are widely used industrially [13]. They are economical but less environmentally friendly. On the other hand, plasma treatment is common, which also improves the hardness and wear resistance of the surface but generates pores that can create areas more prone to corrosion [14,15,16,17].

Among the options that have undergone significant development in recent years, the sol-gel process stands out as a very versatile, environmentally friendly and economically viable synthesis method [18,19]. It basically consists of the chemical reaction of precursors in three successive stages: a first hydrolysis stage, followed by polycondensation and finally, thermal densification.

Starting from a colloidal suspension of very small particles in a solvent, the sol is converted into a gel with a two-phase solid-liquid structure. The precursors used in the sol-gel process can be organic or inorganic. If denser coatings are desired, organic-inorganic hybrids can be prepared to form a more barrier-like layer against solvents [20].

The advantages of using the sol-gel method include the following: the high purity of the products obtained due to the high control in the composition of the precursor materials; no pre-treatment is necessary to cover the metal surface; the synthesis can be carried out at room temperature and at atmospheric pressure; the process has low toxicity and is economical; and the coatings show good chemical resistance, constituting a powerful corrosion inhibitor for metals and alloys. In addition, the intrinsic porosity of sol-gel coatings allows them to host other molecules while preserving their properties, acting as active corrosion protection methods [21].

Various corrosion inhibitors can be added to increase the coating’s protection. Historically, inorganic salts were used (e.g., Ce^3+^ ions [22], La^3+^ ions [23] or Zr^3+^ ions [24]), and later organic molecules and their derivatives that are less toxic and biodegradable. Most of the early organic inhibitors were weak acids, and some of the more recently used ones are molecules containing several heteroatoms, such as amino acids [25,26]. The use of polymers such as polyaniline or polypyrrole as conductive coatings has also been tested. In this case, the protection mechanism is not due to a barrier effect, but it is believed that a passive oxide film is formed on the metal surface by an anodisation process [27,28], which confers the resulting metal/coating system with self-repairing properties and active corrosion protection [29]. Various types of nanoparticles (NPs) have also been tested [30,31]. TiO_2_ NPs have been shown to improve anti-corrosion and antibacterial properties [32]. Graphene and its oxide nanosheets have also been used in sol-gel coatings due to their specific surface areas and impermeability to ions, water, and oxygen. However, they do not provide active protection to the substrate [33,34]. On the other hand, when seeking maximum environmental friendliness, such as toluylalanine they are an effective temporary inhibitor of steel in humid atmospheres [35], and a number of morpholine-Mannich derivatives act as volatile corrosion inhibitors. In the case of seawater, volatile corrosion inhibitors that trap molecules or ions in the medium to prevent their contact with the metal substrate are of interest. Examples of these organic inhibitors that can also be found in the literature are turmeric, L-cysteine or methylene blue, among others [36,37,38]. Some of the organic corrosion inhibitors can, in some cases, offer a double functionality: an efficient response to corrosion phenomena, thanks to the free radicals in their structure, such as turmeric, dimethyl yellow, chlorophenol red or crystal violet with antioxidant power, and the visual perception of pH changes that can identify corrosion processes as they occur, acting as optical sensors. To the best of the authors’ knowledge, this approach has not been developed before.

In this scenario, the aim of this research work is focused on the design, preparation, characterisation, and evaluation of multifunctional sol-gel coatings with different environmentally friendly inhibitors, some of them never used before for this purpose, to be applied on AA2024 aluminium alloy for its corrosion protection.

## 2. Results and Discussion

### 2.1. Coatings Characterisation

Table 1 shows prepared coatings with their corresponding nomenclature, preparation and test conditions.

Coatings thickness was obtained from the interference of reflection spectra in the ultraviolet-visible and near-infrared regions, according to [39]. Table 2 shows specific values for each coating. All coatings had approximate thicknesses between 500 nm and 1 μm.

**Figure 1:**
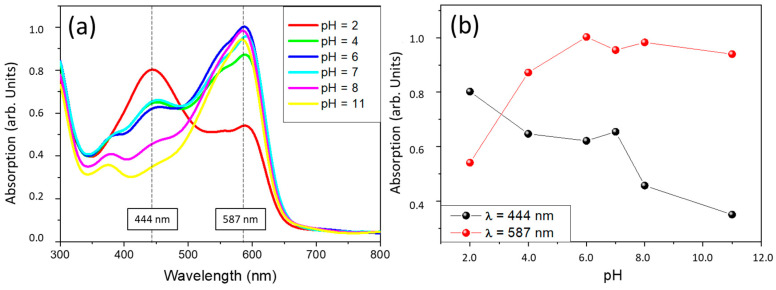
VIS absorption spectra of sample CR on glass substrate at different pHs (**a**). Evolution of maximum absorption spectra at 444 and 587 nm (**b**).

The thickness of the coatings is different due to the different corrosion inhibitors added. The molecules chosen as inhibitors are very different in size and the functional groups they possess. These molecules are responsible for the final pH of the sols and the charge density on the metal, in this case, on the Si ions of the alkoxides. Hydrolysis is facilitated when Si ions are bound to Figure 1 OH groups and inhibited when Si ions are bound to -OR groups. The higher the number of H bound to O, the greater the hydrolysis. In the same way, the literature explains the effect of catalysts. Acidic or basic catalysis influences the hydrolysis and polycondensation rates and thus affects the final gel structure. Acids increase the kinetics of the reaction, and hydrolysis is complete if sufficient water is added. However, high acid concentrations slow down the kinetics of polycondensation and less branched gels are obtained.

Conversely, alkaline or less acidic conditions result in deprotonation of Si-OH ligands forming Si-O- which slows down the rate of hydrolysis, perhaps because the charge density on the Si ion decreases, and the kinetics of the polycondensation reaction increases [40].

In the sols prepared in this work, differences in pHs from 1 to 4 have been observed, although the only difference in the composition of the sols is the added inhibitor (pH of sols DY, CR, and MB could not be measured due to the intense colour of the solution). It seems that the more acidic the pH of the sol initially is, the lower the thickness of the final coating obtained, which is in agreement with what the literature explains about higher hydrolysis kinetics and lower polycondensation kinetics; that is, the more acidic the sol is. As all the coatings were prepared at the same time and left to stir for the same amount of time, the difference in the degree of polymerisation, viscosity and, therefore, the thickness of the coating can only be due to the catalytic effect of the corrosion inhibitor added, maintaining the pH acidic or lowering it, depending on the molecule.

Some of the inhibitors used have a dual function. On the one hand, they act as corrosion inhibitors; on the other hand, they are pH indicators, changing their colour when the environment becomes more alkaline or acidic. These inhibitors are CV, CR, and DY.

The optical behaviour of the coatings as a function of the environmental pH has been studied by immersion in buffers of different pHs for 1 min. CV and DY samples showed overlapped spectra independently of the pH of the solution. Therefore, it seems that these pH indicator molecules that have been introduced into the sol-gel coating lose their functionality when they are encapsulated. The spectra collected for the CR samples are shown in Figure 1. Results show that the CR samples are sensitive to pH changes, and absorption differences can be observed in the visible region. The molecules of chlorophenol red would act as pH sensors despite being encapsulated within the sol-gel coating and would not lose their properties as pH indicators.

In the case of sample CR, two absorption maxima are observed at 444nm and 587nm at an initial pH = 2, corresponding to violet and yellow wavelength absorption, respectively, which corroborates the visually observed colours. It turns from a reddish colour to an orange-yellow with decreased pH.

**Figure 2:**
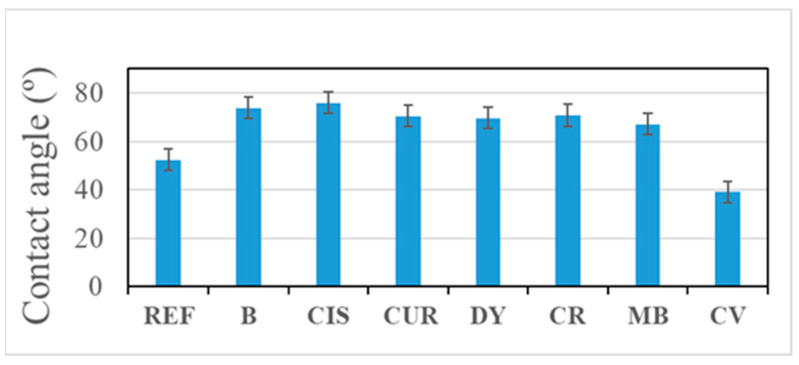
Contact angle values of the samples.

Contact angle measurements show a moderate hydrophilic behaviour for all coatings (Figure 2). All obtained values are less than 90°. Lower values have been obtained for the sample REF, which is the uncoated Al alloy, and for the sample CV. The angle between the water and the CV surface is relatively low, so the area in contact with the water is higher in this case, which could be detrimental because water droplets are more likely to remain on the surface. The best results are obtained with B, CIS, and CR samples.

### 2.2. Weathering Test

At the beginning of the corrosion test, the pH of all 0.6M NaCl solutions was neutral, but after 14 days of sample immersion, the pH of almost all solutions changed to acidic, pH was around 4, or even lower. In samples solution DY and CV, the pH after the test was around 3. This result indicates that the medium has acidified as the corrosion process of the alloys has been carried out, so it seems that protons have been released to the saline medium, and the following reaction may have happened.

↓ Al + 3H_2_O → ↓ Al(OH)_3_ + 3 H^+^ (1)

**Figure 3:**
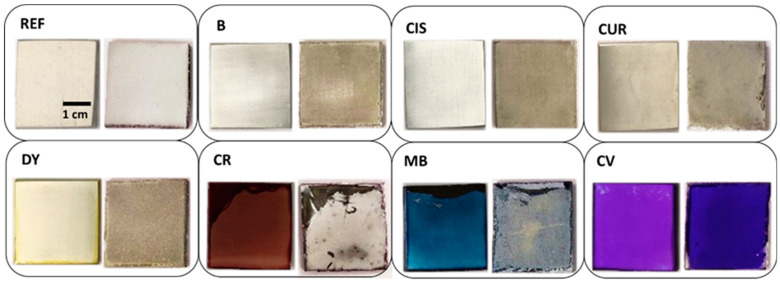
Overview picture of sol-gel coated samples before (**left**) and after (**right**) the corrosion test.

All samples show signs of surface deterioration after two weeks of immersion in the corrosive medium. Figure 3 shows the comparison of the samples before and after the corrosion test. Some of them show signs of corrosion, as the REF sample is fully covered with white deposits of oxides well adhered to the surface. Part of the aluminium in the alloy may have been dissolved, as indicated in Equation (1), thus partially compensating the weight loss due to Al dissolution by the formation of Al(OH)_3_ deposits. Thus, it may explain the lightweight variation of only some milligrams in the samples after the weathering test. On the bottom of some of the sample containers, small black dots are observed together with abundant white crystallisations, which could confirm these statements.

### 2.3. Optical Microscopy Characterisation

**Figure 4:**
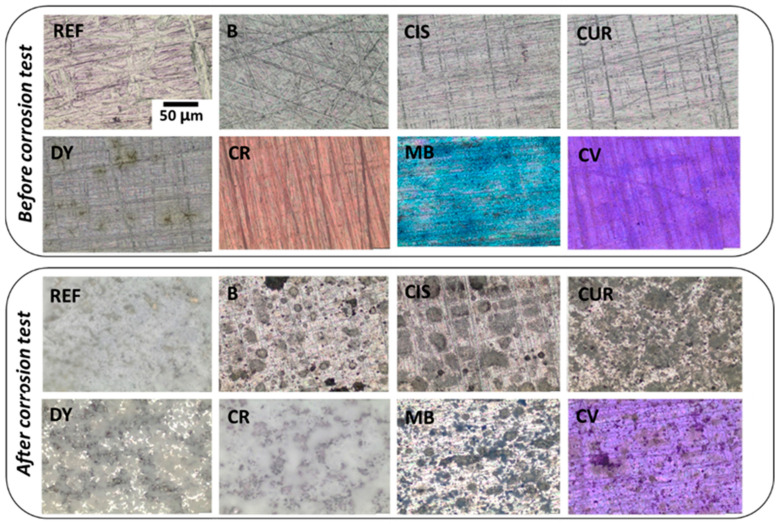
OM images of the sol-gel coated samples: before and after the corrosion test.

Optical microscopy images of the surface of the samples before and after the corrosion test have been taken to monitor microstructural changes (Figure 4). Before the test, micrographs of the surfaces show homogeneous and transparent coatings colourless for samples B, CIS, CUR, and DY, a red coating for sample CR, blue for sample MB, and violet for sample CV. The polishing lines of the surfaces are also visible. Once the corrosion test was completed, samples were rinsed three times with distilled water to remove surface deposits, and they were again optically characterised.

In the sample without inhibitor (B) and the samples with curcumin (CUR), L-cysteine (CIS), methylene blue (MB), and crystal violet (CV), localised corrosion is observed, which can be in the form of pitting between the grains of alloy AA2024 and oxides resulting from the reaction with the corrosive medium. As for the sample without sol-gel (REF), the sample with dimethyl yellow (DY) and the sample with chlorophenol red (CR), a more uniform distribution of these corrosion products is observed, forming a thick corrosion layer.

### 2.4. Weight Variation

**Figure 5:**
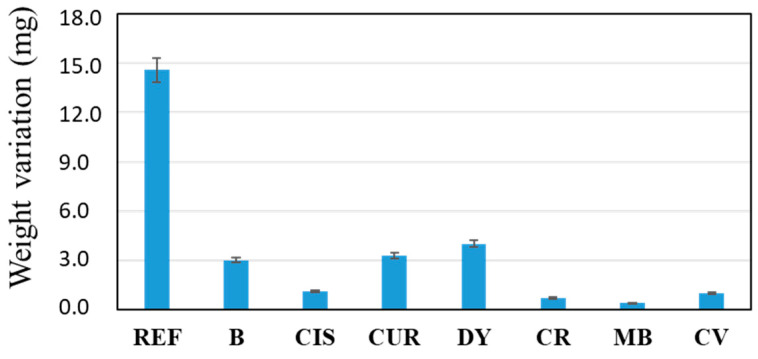
Difference in weight of samples before and after the corrosion test.

Each sample was weighed before and after the corrosion test. Once the samples were washed with distilled water three times after the test, they were placed in a desiccator and weighed one week later so that all the samples would lose their residual moisture after the 14 days of testing and washing. Figure 5 shows the weight variation of each sample subjected to the corrosion test (before and after the test). The weight variation is the final weight minus the initial weight.

An increase in the weight of the samples before and after the corrosion test, in the order of milligrams, is observed. On the one hand, a certain reduction in the weight of the sample was expected due to the loss of metal released by reacting with the ions in the medium. On the other hand, the same metal ions have most likely reacted with OH- ions, forming metal hydroxides deposited on the surface again, increasing the final weight of the samples.

In the weight variation, the uncoated sample (REF) stands out with an increase of almost 15 mg in weight. On the other hand, the rest of the samples can be grouped into two blocks, i.e., those that have barely modified their weight (<2 mg), CIS, CR, MB and CV; on the other hand, those that are in an intermediate situation between the REF sample and the previous ones: B, CUR and DY. A priori, it is logical to think that the thicker the coating, the greater its protective effect on the alloy. However, sol-gel coatings have an inherent nanoporosity in their structure and do not exert a barrier effect, such as other organic polymers (i.e., epoxy resins or polyurethanes). In the coatings presented in this work, the corrosion-inhibiting effect of the chosen organic molecules must also be taken into account. Thus, the CUR and DY coatings, although they have a lower thickness than the sol-gel coating without inhibitor, sample B, the weight variation observed in Figure 5 is similar, so it is thought that their protective effect is also similar. The coatings of similar thickness to the B sample (reference sample coated but without inhibitor), such as the CIS, CR, MB, and CV samples, show significantly better results against corrosion. Thus, it seems that the thickness of the coating affects the barrier effect, and furthermore, the addition of inhibitor molecules improves the anti-corrosion effect of the coating. The presence of heterogroups and double bonds in the inhibitor molecules seems to play a crucial role in the corrosion inhibitory effect of the coatings.

### 2.5. Surface Characterisation by SEM

**Figure 6:**
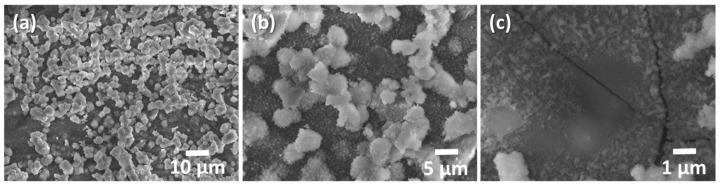
SEM images of the surface of sample REF. (**a**) 10 μm; (**b**) 5 μm; (**c**) 1 μm.

SEM/EDX characterisation allowed a compositional and morphological study of the corrosion products, the surface of the metal alloy, and the sol-gel coatings. Figure 6 shows the surface of sample REF in secondary electrons (SE), corresponding to the alloy subjected to the ageing test without sol-gel coating. It shows a completely covered surface with white deposits. At higher magnification (Figure 6c), some cracks appear on the surface of the alloy.

**Figure 7:**
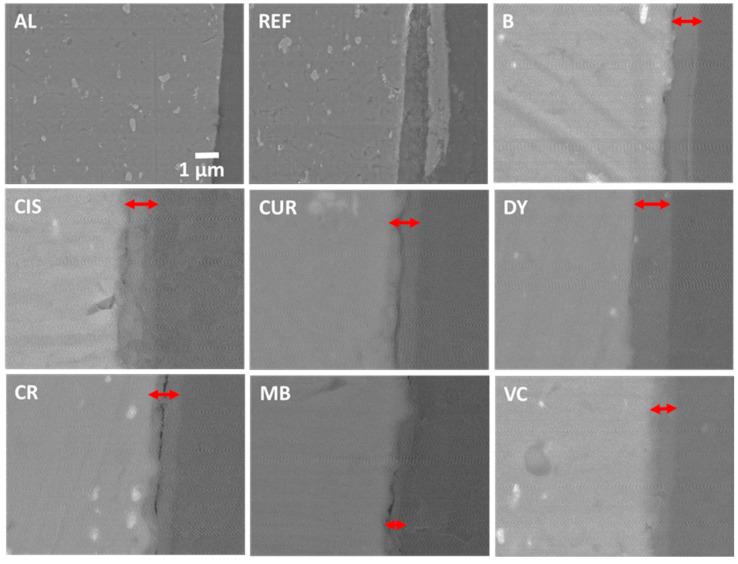
SEM images of a crosssection of samples AL, REF, B, CIS, CUR, DY, CR, MB, and CV.

An EDX analysis of the surface of Figure 6a shows exclusively O and Al, probably in the form of Al oxides or hydroxides. If these deposits had good adhesion with the substrate, they could act as protectors, but in this case, part of the product is easily lifted, which may indicate that they could be mostly hydroxides. Similarly, the samples that have been coated with the sol-gel are shown in backscattered electrons (BSE) in Figure 7 [41,42]. In comparison, no cracks or corrosion products such as oxides/hydroxides appear in the sample without protective coating from the crosssection view (sample AL of Figure 7). Sample REF, corresponding to the weathered alloy, shows flakes partially detached from the bulk (sample REF in Figure 7). All coated samples show the sol-gel coatings deposited on the alloys. The lighter and brighter grey colour of the SEM images corresponds to the aluminium alloy substrate (left side of the images). The sol-gel coating with the corresponding inhibitor is marked with a red double arrow on all images, and the darkest grey colour corresponds to the organic epoxy resin of the embedding (right side of the images).

The coatings have remained unaltered during the accelerated salt weathering and are visible in the SEM images. Their thicknesses have remained constant and in good agreement with the values obtained from the VIS reflectance measurements (Table 2). EDX analyses of the images show a composition similar to that of the nominal AA2024 alloy for the inside of the REF sample, where the O content increases when approaching the outer crust (Table 3).

The analyses of the sol-gel coatings from the crosssection of samples in Figure 7 shows the presence of Si and O in the coating and C from the organic part of the precursor alkoxides and the inhibitors. Variable amounts of Cl can also be observed, probably from the NaCl solution in which the samples have been immersed and have not been removed by the washes with distilled water. Finally, small amounts of Al appear from the substrate. No major differences are observed between the sol-gel coatings with the different inhibitors.

## 3. Conclusions

Seven hybrid sol-gel coatings with different corrosion inhibitors have been prepared, applied on AA2024 alloy samples, and characterised before and after a corrosion test in a simulated saline environment. The prepared coatings have proved to be effective against corrosion phenomena in a saline environment, and the observed corrosion was more severe in the uncoated sample. In this sample, numerous whitish deposits were visible on its surface, and some cracks when approaching a neat area. On the coated samples, no cracks or very significant deterioration were observed.

Weight variations of tens of milligrams before and after the corrosion test are measured in the uncoated sample, and slight increases of several milligrams for the coated samples so that the coating plays a fundamental role in inhibiting the formation of superficial aluminium hydroxides.

The thicknesses of the coatings calculated by UV-VIS reflectance spectra vary from 500 nm to 1 μm and are of the same order as the ones observed in the SEM images of the crosssections of the samples subjected to the corrosion test. This demonstrates the effectiveness of the coating and the absence of deterioration. The addition of a different corrosion inhibitor molecule to a sol of the same composition varies the pH of the solution and the charge density of the Si^4+^ ions of the alkoxides. This fact varies the speeds of the hydrolysis and polycondensation reactions, which are accelerated or slowed down. Therefore the viscosity of the sol at the time of its application by dip-coating may be different, thereby obtaining coatings of different thicknesses by varying the inhibitor molecule of the sol-gel coating.

When comparing the results of the coated samples with the selected inhibitors (CIS, CUR, DY, CR, MB, and CV) and the sample coated only with sol-gel (B), with optical microscopy, it has been possible to confirm the reduction of the hydroxides and other corrosion products deposited. This may be due to plugging the pores with the inhibitor when the corrosion process begins on the substrate. The weight increase of the coatings after the test is very small for the CIS, CR, MB, and CV samples, confirming its effectiveness in inhibiting degradation on the surface of the AA2024 alloy. However, there is no direct relation between the effectiveness of protection against corrosion and the coating thickness. This is because corrosion protection is achieved through active protection due to the action of the inhibitors and not only due to a barrier effect. Additionally, the CR coating, which has been optically characterised in pH buffer solutions, has demonstrated a dual functionality as a pH indicator and corrosion inhibitor. The colour change as a function of pH variation is visible by UV-VIS spectrophotometry and to the naked eye, which is a very significant result for future industrial applications.

## 4. Materials and Methods

### 4.1. Aluminium Alloys

Commercial AA2024 aluminium alloy (Cu 4.5 and Mg 1.5 in wt%) was selected as a substrate for this work. Plane sheets with a thickness of 0.30 cm were cut into squares of 3 × 3 cm^2^, exposing a total area of 21.6 cm^2^. They were all polished with 1200 SiC paper, rinsed with ethanol and dried at 25 °C prior to coating.

### 4.2. Sol-Gel Coatings

Tetraethyl orthosilicate (TEOS, Sigma Aldrich, 99%), 3-(trimethoxysilyl) propyl methacrylate (Sigma Aldrich, 98%) and 3-(cloropropil)trietoxisilano (Sigma Aldrich, 95%) were used as precursors (molar ratio 2:1:1). Absolute ethanol was employed as solvent. Distilled water was added to promote the complete hydrolysis of the alkoxides, and nitric acid was added as a catalyst. The molar ratio of precursors: EtOH:H_2_O:HNO_3_ was 1:8:3:0.01.

**Figure 8:**
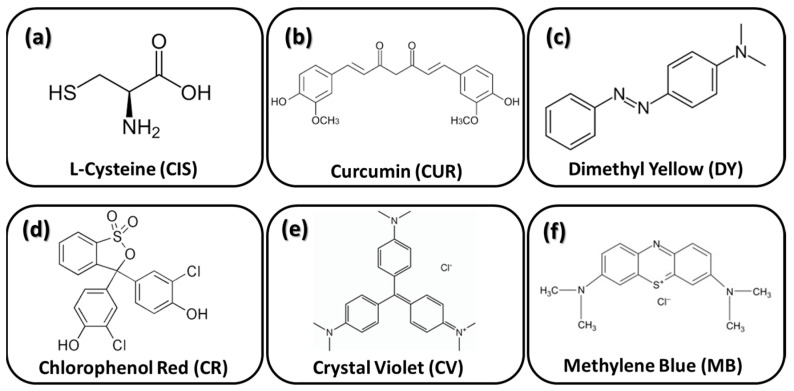
Corrosion inhibitors added into the sol-gel coatings: (**a**) L-cysteine; (**b**) curcumin; (**c**) dimethyl yellow; (**d**) chlorophenol red; (**e**) crystal violet; and (**f**) methylene blue.

Prior to the sol hydrolysis process, an environmentally friendly corrosion inhibitor was added to the sol. For this purpose, six organic molecules have been selected, i.e., L-cysteine (CIS), curcumin (CUR), dimethyl yellow (DY), chlorophenol red (CR), crystal violet (CV) and methylene blue (MB) (Figure 8).

The inhibitors were chosen due to their properties as biocompatible molecules, non-toxic and environmentally friendly. Specifically, molecules with conjugated bonding and heteroatoms have been shown to be more effective as corrosion inhibitors [43].

Coatings were performed by dip-coating on AA2024 aluminium alloy and commercial glass slides at a withdrawal rate of 5 mm·s^−1^. Final thermal treatment was performed at 80 °C for 24 h to complete the densification of the resulting coatings.

### 4.3. Corrosion Test Solution

To evaluate the effectiveness of the coatings doped with several molecules after their application to the Al alloy, a corrosion test in a 0.6 M NaCl solution simulating marine medium was employed [44]. Samples suspended on a fishing line were immersed into new and sterilised single hermetic containers of 150 mL for 14 days and stored in a UV hood [45]. UV irradiation was considered sufficient to prevent bacterial growth, and the salt solution was not changed periodically during the duration of the corrosion test [46]. At the end of the test, samples were washed 3 times by immersion in distilled water. The weight of the coated samples before and after the corrosion test was registered.

### 4.4. Characterisation Techniques

The macroscopic information of the coatings surfaces on the Al alloy before and after the degradation test was obtained by optical microscopy (OM) in reflection mode with an Olympus CX41 optical microscope.

UV-VIS optical absorption spectra of the coatings applied on glass slides at different pHs were registered with a Shimadzu UV-3100 (UV VIS-NIR) spectrophotometer. It is equipped with an integrating sphere which enables reflection measurements that allow the measurement of the thickness of the coating.

Contact angle measurements were carried out to determine the hydrophilic or hydrophobic behaviour of the different coatings. A Kruss EasyDrop shape analyser was used at room temperature. Doses of 2µL of distilled water were used for each measurement.

Surface characterisation and semi-quantitative chemical composition analyses of the corroded surfaces and small deposits that remained adhered to the surface of the samples were carried out by SEM with an electronic microscope model JEOL JSM 6335F operating at an acceleration voltage of 20 kV. An energy-dispersive X-ray (EDX) detector was employed for the elemental analysis of the surfaces. All samples were previously coated with 5 nm of Au to avoid charging effects.

## Figures and Tables

**Table 1:** Description of the prepared samples.

Sample Name	Sol-Gel Coating	Corrosion Inhibitor Added	Corrosion Test Performed
AL	NO	NO	NO
REF	NO	NO	YES
B	YES	NO	YES
CIS	YES	L-cysteine	YES
CUR	YES	Curcumin	YES
DY	YES	Dimethyl Yellow	YES
CR	YES	Chlorophenol Red	YES
MB	YES	Methylene Blue	YES
CV	YES	Crystal Violet	YES

**Table 2:** Coatings thickness.

Sample	B	CIS	CUR	DY	CR	MB	CV
Thickness (nm)	(104 ± 15)·10^1^	(102 ± 15)·10^1^	514 ± 95	718 ± 52	(91 ± 10)·10^1^	874 ± 56	719 ± 54

**Table 3:** EDX analysis of the crosssection of the weathered samples.

Sample	C (wt. %)	O (wt. %)	Mg (wt. %)	Al (wt. %)	Si (wt. %)	Cl (wt. %)	Cu (wt. %)
REF int		6.1 ± 1.2	1.38 ± 0.28	89.1 ± 1.4			3.41 ± 0.81
B coat	58.5 ± 2.9	23.3 ± 2.7	-	7.84 ± 0.69	6.04 ± 0.60	4.32 ± 0.51	-
CIS coat	37.5 ± 2.6	42.1 ± 2.2	-	16.78 ± 0.91	1.43 ± 0.28	2.15 ± 0.28	-
CUR coat	64.1 ± 2.1	19.6 ± 2.0	-	6.68 ± 0.46	5.93 ± 0.45	3.72 ± 0.35	-
DY coat	61.7 ± 2.5	21.3 ± 2.4	-	2.91 ± 0.32	8.24 ± 0.62	5.86 ± 0.50	-
CR coat	58.7 ± 2.7	26.7 ± 2.5	-	5.64 ± 0.51	5.23 ± 0.49	3.66 ± 0.42	-
MB coat	63.4 ± 1.8	23.2 ± 1.7	-	5.56 ± 0.35	4.85 ± 0.34	2.93 ± 0.26	-
CV coat	57.6 ± 2.5	25.5 ± 2.3	-	6.39 ± 0.51	6.28 ± 0.52	4.25 ± 0.42	-

The “int” means the interior of the substrate; the “coat” means the coating.

## Data Availability

Not applicable.
